# Using Inclusive Design to Develop a Tele–Tai Chi Intervention for Older Adults With Long-Term Mobility Disability

**DOI:** 10.1093/geroni/igaa057.2018

**Published:** 2020-12-16

**Authors:** Tracy Mitzner, Elena Remillard, Kara Cohen, Jordan Chen

**Affiliations:** 1 Georgia Institute of Technology, Atlanta, Georgia, United States; 2 Georgia Institute Of Technology, Atlanta, Georgia, United States

## Abstract

Tele-technologies may be able to increase access to evidence-based exercise interventions for adults aging with long-term mobility disabilities. This population experiences substantial barriers in attending such programs in person, including lack of transportation to classes, inaccessible buildings where classes are held, and lack of appropriate modifications offered for this population of older adults. It is critical to overcome such barriers to ensure this population has an opportunity to receive the benefits of evidence-based programs. In this study we are translating an in-person evidence-based tai chi intervention, Tai Chi for Arthritis, to an online platform using videoconferencing software for those aging with long-term mobility disabilities. We will describe our approach of including users from the target population and industry representatives (videoconferencing software developer, Tai Chi for Arthritis program developer as well as local master trainer) in the adaptation of the intervention and present the key findings from doing so.

